# Damage to the Biliary Tree as a Result of Laparoscopic Cholecystectomy (Paper Discussion)

**DOI:** 10.1155/2000/29527

**Published:** 2000-08


*HPB Surgery*, 2000, Vol. 11, pp. 423-430
Reprints available directly from the publisher
Photocopying permitted by license only

© 2000 OPA (Overseas Publishers Association) N.V.
Published by license under
the Harwood Academic Publishers imprint,
part of The Gordon and Breach Publishing Group.
Printed in Malaysia.

**HPB INTERNATIONAL**

**EDITORIAL & ABSTRACTING SERVICE**

JOHN TERBLANCHE EDITOR
Department of Surgery,
Medical School · Observatory 7925
Cape Town · South Africa
Telephone: 27-21-406-6204
Telefax 27-21-448-6461
E-mail: JTER BLAN@UCT GSHI.UCT.AC.2A

### Damage to the Biliary Tree as a Result of Laparoscopic Cholecystectomy (Paper Discussion)*

### ABSTRACT

*William S. Richardson and John G. Hunter*[†]

*Keywords:* Cholecystectomy, laparoscopic cholecystectomy, bile duct injury

### DISCUSSION

This paper, by Bergman *et al.*, is a great compendium of work that properly discusses the management of biliary injuries. Bile duct injuries are the greatest fear of the laparoscopic surgeon and although they occur less frequently than in the past, they are still reported in 0.2% of cases. Even in the hands of a skilled laparoscopic surgeon there are more bile duct injuries than during open surgery. The best management requires a team skilled in handling these injuries.

The best solution is avoidance. There are five steps in avoiding bile duct injuries: (1) use a 30 degree laparoscope in order to see over the duodenum; (2) retract the fundus of the gallbladder firmly over the liver and cephalad in order to remove the redundancy of the cystic duct; (3) retract the infundibulum of the gallbladder to the patient's right separating the cystic duct from the common duct; (4) after positive identification of the cystic duct confirm it by dissecting all the way to the infundibulum of the gallbladder before transection; (5) perform cholangiography routinely.

Another rule of thumb is that if more than one package of hemoclips (6 clips) has been used, indicating a difficult dissection, the risk of bile duct injury will be higher. Also, a surgeon should never allow poor visual quality while dissecting. Techniques for safe performance of laparoscopic cholecystectomy are now well known. Thus, the rate of bile duct injury has decreased.

Routine use of cholangiogram is not advocated by all surgeons, perhaps because of the rare occurrence of common bile duct stones.

*Bergman, J.J.G.H.M., Van Den Brink, G.R., Rauws, E.A.J., DeWit, L., Obertop, H., Huibregtse, K., Tytgat, G.N.J. and Gouma, D.J., Treatment of Bile Duct Lesions After Laparoscopic Cholecystectomy. Gut 1996; 38, 141-147.

[†]Corresponding author.

The argument is that when liver function tests are normal the added time and potential morbidity with cholangiogram is not warranted. We use cholangiography not to find common duct stones but as a tool for detecting bile duct injuries which may have occurred during laparoscopic cholecystectomy. In our series of 177 patients referred with bile duct injuries, 104 patients did not have cholangiography and therefore did not have the advantage of early repair of injury. Early repair may lessen morbidity because of less inflammation, and fibrosis. However, if the extent of injury is not known such as the extent of ischemic injury to the bile duct, it is best to wait until the bile duct has healed prior to surgical therapy since the risk of duct stenosis may be high after early repair in this instance.

The algorithm proposed by Bergman *et al.*, is excellent and should be advocated. Bile duct injury is suspected when abdominal pain, bile fistula, jaundice or fever are present. Liver function studies and abdominal ultrasound or CT scan should be performed. We have not found HIDA scans to be of much value in the work-up of bile duct injuries. Some authors would suggest transhepatic cholangiogram (THC) primarily, and ERCP secondarily. Also, some advocate routine fistulaogram but others say this has a high risk of cholangitis. A fistulogram does identify anatomy without the need for ERCP or THC, however fistulogram is not a therapeutic modality.

There have been many ways to classify bile duct injuries and classification has changed to coincide with the treatments of complications. The classification proposed is workable and resembles those used in most recent studies. Most cystic duct or common duct leaks can be stented by THC or ERCP and will close in six weeks to two months. Bile duct transections and resections usually require a Roux-en-Y anastomosis or choledochoduodenectomy for diversion, although primary duct repair over a T-tube has also been described. If not immediate, repair should be done several months after injury to allow healing and softening of adhesions. This requires stenting of the hepatic ducts using THC. After injury to the common bile duct during laparoscopic cholecystectomy the ducts under the Hilar plate are spared. The surgeon should be accustomed to operating in this area in order to do the anastamosis. Bile duct stenoses are managed first by stenting. Some groups such as Bergmen *et al.*, have had success with stenting alone, using routine stent changes over a long period leading to resolution of the structure. This is not universally accepted and many authors advocate stenting followed by definitive surgical repair several months after injury.

Strategies for avoidance of bile duct injuries are becoming quite well known but even in the best of hands they still occur. If a bile duct injury occurs, early recognition and management by a medical team skilled in bile duct repair will decrease the risk of further complications. There are still controversies regarding repair of duct injuries but large series, like Bergman *et al.*'s will help define the best management strategy.

### *References*

Hunter J. G. (1991) Avoidance of bile duct injury during laparoscopic cholecystectomy. *Am. J. Surg.*, **162**, 71–6.

Soper, N. J., Flye, M. W., Brunt, L., Stockmann, P. T., Sicard, G. A., Picus, D., Edmundowicz, S. A. and Aliperti, G. (1993) Diagnosis and management of biliary complications of laparoscopic cholecystectomy. *Am. J. Surg.*, **165**, 663–9.

Voyles, C. R., Sanders, D. and Hogan, R. (1994) Common bile duct evaluation in the era of laparoscopic cholecystectomy 1050 cases later. *Ann. Surg.*, **219**, 744–50.

